# The Predictive Value of Radiomics for Esophagotracheal Fistula after Radiotherapy in Esophageal Cancer

**DOI:** 10.2174/0115734056452685260414044447

**Published:** 2026-04-21

**Authors:** Huiyao Chen, Yanglong Wu, Congcong Wu

**Affiliations:** 1 Department of Pathology, The Second Affiliated Hospital and Yuying Children's Hospital of Wenzhou Medical University, Wenzhou, Zhejiang Province, China; 2 Wenzhou Medical University Renji College, Wenzhou, Zhejiang Province, China; 3 Oncology Department of Radiotherapy and Chemotherapy, The Second Affiliated Hospital and Yuying Children's Hospital of Wenzhou Medical University, Wenzhou, Zhejiang Province, China

**Keywords:** Radiomics, Esophageal cancer, Esophagotracheal Fistula, Radiotherapy, CT images, Intensity modulated radiotherapy (IMRT)

## Abstract

**Introduction:**

Esophagotracheal Fistula (ETF) is a serious complication following radiotherapy for esophageal cancer, with treatment outcomes significantly worse than expected.

**Methods:**

Pre-radiotherapy CT images and clinical data from patients with esophageal malignancies treated at the Second Affiliated Hospital of Wenzhou Medical University between January 2015 and December 2023 were retrospectively analyzed. Tumor contours were manually delineated using 3D Slicer, and radiomic features were extracted using PyRadiomics. Features associated with ETF development (*p*<0.05) were identified via the Mann-Whitney U test and further refined using Least Absolute Shrinkage and Selection Operator (LASSO) regression to determine the final radiomic signature. Subsequently, univariate and multivariate binary logistic regression analyses were performed.

**Results:**

The study included 77 patients, 30 of whom developed ETF. Of the initial 845 radiomic features, 10 were significantly associated with ETF. Among clinical factors, the type of radiation therapy was an independent predictor for ETF. In the training cohort, the radiomics model achieved an AUC of 0.866 (95% CI: 0.7907–0.9402), with a sensitivity of 0.831 and specificity of 0.792. The combined model (radiomics + clinical features) achieved an AUC of 0.892 (95% CI: 0.8238–0.9601), sensitivity of 0.823, and specificity of 0.912. In the validation cohort, the radiomics model had an AUC of 0.736 (95% CI: 0.5781–0.8947), sensitivity of 0.833, and specificity of 0.621. The combined model achieved an AUC of 0.791 (95% CI: 0.6461–0.9354), sensitivity of 0.822, and specificity of 0.797.

**Discussion:**

The combination of radiomic and clinical features achieves excellent AUC performance and shows potential for the non-invasive prediction of ETF following radiotherapy in esophageal cancer patients.

**Conclusion:**

The model, combined with radiomic and clinical features, has great predictive value.

## 1 INTRODUCTION

Esophageal cancer is a leading cause of cancer-related mortality worldwide. In 2022, there were an estimated 510,716 new cases and 445,129 deaths, ranking it 11th and 7th among malignancies, respectively [1, 2]. Radiation Therapy (RT) is a cornerstone in the management of thoracic tumors; however, it is associated with adverse events, some of which can be life-threatening. Esophagotracheal Fistula (ETF) is a rare but severe complication of esophageal cancer, primarily arising from tumor invasion through the esophageal wall into surrounding structures, leading to necrosis. An ETF allows gastrointestinal contents to enter the airway, leading to severe infection, abscess formation, or hemorrhage. Consequently, the prognosis for patients with ETF is extremely poor, with a median survival of only 2–3 months [3, 4]. Early prediction of ETF could therefore help mitigate mortality risk and improve patient outcomes.

Medical imaging serves as a non-invasive tool widely used in cancer diagnosis, treatment planning, and monitoring. Radiomics, which converts medical images into quantitative data, offers an objective and precise approach to support clinical decision-making [5]. For instance, voxel-level radiomic and deep learning models show high accuracy in predicting pathologic complete response to neoadjuvant immunochemotherapy in esophageal squamous cell carcinoma [6]. Furthermore, integrating radiomics with cell-free DNA can enhance models predicting overall survival and treatment response in esophageal adenocarcinoma [7]. However, research applying radiomics to predict post-radiotherapy ETF remains limited. Previous studies have mainly focused on conventional tumor characteristics such as length, wall thickness, volume, and ulcer depth [8-10].

Given the widespread availability of CT imaging, the study aimed to use machine learning to identify radiomic features from contrast-enhanced CT scans that correlate with ETF risk. By combining these features with clinical parameters, a predictive model for ETF was sought to be developed, enabling improved risk stratification and monitoring of esophageal cancer patients following radiotherapy.

## 2 METHODS

### 2.1 Patient Selection

This retrospective study included patients with esophageal squamous cell carcinoma who received treatment at the Second Affiliated Hospital of Wenzhou Medical University between January 2015 and December 2023. The study was conducted in accordance with the Declaration of Helsinki and was approved by the institutional ethics committee, which waived the requirement for informed consent (approval numbers: 2024-K-100-01, 2024-K-100-02, Second Affiliated Hospital of Wenzhou Medical University, China).

Inclusion criteria were: (a) pathological confirmation of esophageal squamous cell carcinoma; (b) treatment with curative or palliative radiotherapy/chemoradiotherapy; (c) availability of complete pre-radiotherapy contrast-enhanced chest CT images.

Exclusion criteria were: (a) multiple primary esophageal cancers or concurrent thoracic malignancies; (b) prior thoracic radiotherapy; (c) previous esophageal surgery; (d) ETF due to non-malignant causes (*e.g*., congenital or traumatic); (e) fistula present before radiotherapy initiation.

The case group comprised all patients diagnosed with ETF between 2015 and 2023, confirmed via endoscopy, CT, esophagography, or clinical assessment. The control group consisted of randomly selected patients without ETF, matched to the case group at a ~1:1.5 ratio. Collected clinical data included age, gender, tumor location, radiotherapy type and total dose, chemotherapy status, hypertension, diabetes, and smoking history.

### 2.2 Image Omics Feature Extraction and Statistical Analysis

Tumor Volumes of Interest (VOIs) were manually contoured on pre-radiotherapy CT scans by a junior radiation oncologist and reviewed by a senior specialist using 3D Slicer software (version 4.11.1, https://www.slicer.org). A representative CT image with tumor contour is shown in Fig. (**1**). No additional image preprocessing was performed. Radiomic features were extracted using PyRadiomics.

### 2.3 Statistical Analysis and Model Development

Patients were randomly split into a training cohort (70%) and a validation cohort (30%). Radiomic features significantly associated with ETF (*p*<0.05, Mann-Whitney U test) in the training set were subjected to LASSO regression for feature selection and dimensionality reduction. The selected features were linearly combined, weighted by their respective LASSO coefficients, to generate a radiomics score (Rad-score) for each patient.

Univariate and multivariate Cox regression analyses were used to identify clinical predictors of ETF. Two predictive models were constructed: a radiomics-only model and a combined model incorporating the Rad-score and significant clinical factors. A nomogram was developed based on the combined model. Model performance was evaluated using receiver operating characteristic (ROC) curves, with the Area Under the Curve (AUC) quantifying discrimination. Decision Curve Analysis (DCA) assessed clinical utility.

Statistical analyses were performed using R (version 3.6.2). Between-group comparisons for clinical variables utilized the Chi-square test, independent t-test, or Mann-Whitney U test in SPSS, as appropriate. A *p*-value <0.05 was considered statistically significant.

## 3 RESULTS

### 3.1 Clinical Characteristics

The study comprised 77 patients (71 males and 6 females), who were divided into a non-ETF control group (n=47) and an ETF case group (n=30). Among the ETF cases, 29 fistulas were located in the trachea and one in a bronchus. The mean ages were 64.4 and 65.3 years, and the mean radiation doses were 4744 cGy and 5183 cGy for the case and control groups, respectively. Detailed clinical features are presented in Table **1**. The training and validation cohorts comprised 54 and 23 patients, respectively, with no significant differences in clinical characteristics between them.

### 3.2 Model Construction and Validation

A total of 845 radiomic features were extracted per patient. Initial screening in the training cohort identified 386 features associated with ETF (*p*<0.05). LASSO regression selected ten key features to build the Rad-score, calculated as follows:

Radscore=(3.905973e+00)+originalshapeMaximum2DDiameterColumn*(6.239942e-03)+originalgldmSmallDependenceLowGrayLevelEmphasis*(-3.518082e+02)+wavelet.LLHfirstorder90Percentile*(-1.411244e-02)+wavelet.LLHfirstorderSkewness*(-1.350767e-01)+wavelet.LLHglrlmRunLengthNonUniformityNormalized*(-4.560903e-02)+wavelet.HLHglszmLargeAreaLowGrayLevelEmphasis*(1.532300e-06)+wavelet.HHHfirstorderSkewness*(-2.426095e-01)+wavelet.HHHglrlmRunEntropy*(1.233795e+00)+wavelet.LLLfirstorder10Percentile*(2.679849e-03)+wavelet.LLLglcmInverseVariance*(8.718345e-02).

Univariate analysis identified radiation dose, radiation type, and Rad-score as significant risk factors for ETF (*p*<0.05). Multivariate analysis confirmed radiotherapy type and Rad-score as independent predictors (Table **2**).

A nomogram incorporating these independent predictors was developed Fig. (**2**). Performance metrics for both models are shown in Fig. (**3**). In the training cohort, the radiomics model achieved an AUC of 0.866 (95% CI: 0.7907–0.9402), sensitivity of 0.831, and specificity of 0.792. The combined model achieved an AUC of 0.892 (95% CI: 0.8238–0.9601), sensitivity of 0.823, and specificity of 0.912. In the validation cohort, the radiomics model had an AUC of 0.736 (95% CI: 0.5781–0.8947), sensitivity of 0.833, and specificity of 0.621, while the combined model achieved an AUC of 0.791 (95% CI: 0.6461–0.9354), sensitivity of 0.822, and specificity of 0.797. Decision curve analysis demonstrated the favorable clinical utility of the combined model Fig. (**4**).

## 4 DISCUSSION

This study presents a practical model combining CT-based radiomics and clinical features to improve the prediction of ETF in esophageal cancer patients after radiotherapy, offering a new approach for risk stratification.

The underlying biological aggressiveness of esophageal cancer is likely a fundamental driver of ETF. Previous studies have identified clinical risk factors, including advanced T-stage, tumor stenosis, age, and specific chemotherapy regimens [11, 12]. Patients with T4 disease are at higher risk, possibly due to deep invasion impairing normal tissue repair during radiotherapy [13]. Re-irradiation also increases fistula risk, warranting caution in patients with T4 or extensive nodal disease [14]. Nutritional and immune status markers (*e.g*., low ECOG PS, serum cholesterol, BMI) have been linked to impaired tissue healing [15, 16], but robust predictive biomarkers for ETF are still lacking. Radiomics may bridge this gap by quantifying macroscopic image patterns that reflect underlying tumor biology and microenvironment.

While numerous radiomics studies have focused on predicting treatment response or survival in esophageal cancer [17, 18], few address ETF prediction. Integrating voxel-level features with deep learning allows the capture of rich textural and morphological information, revealing tumor heterogeneity and potentially guiding therapy [19]. Models combining multiple imaging modalities or integrating radiomics with clinical data typically outperform single-source models [20, 21]. Our study contributes a focused radiomics investigation specifically targeting ETF.

## 5 STUDY LIMITATION

This study has limitations. Its single-center, retrospective design may limit generalizability. Direct comparisons between specific treatment regimens were not performed. Future work should aim to incorporate larger, multi-center datasets and prospective validation to enhance robustness and diversity.

## CONCLUSION

The combination of radiomic and clinical features enables effective non-invasive prediction of ETF risk in esophageal cancer patients prior to radiotherapy. This integrated model demonstrates potential for clinical application in risk stratification and personalized treatment planning, though external validation in larger multicenter cohorts is warranted to confirm its generalizability.

## Figures and Tables

**Fig. (1):**
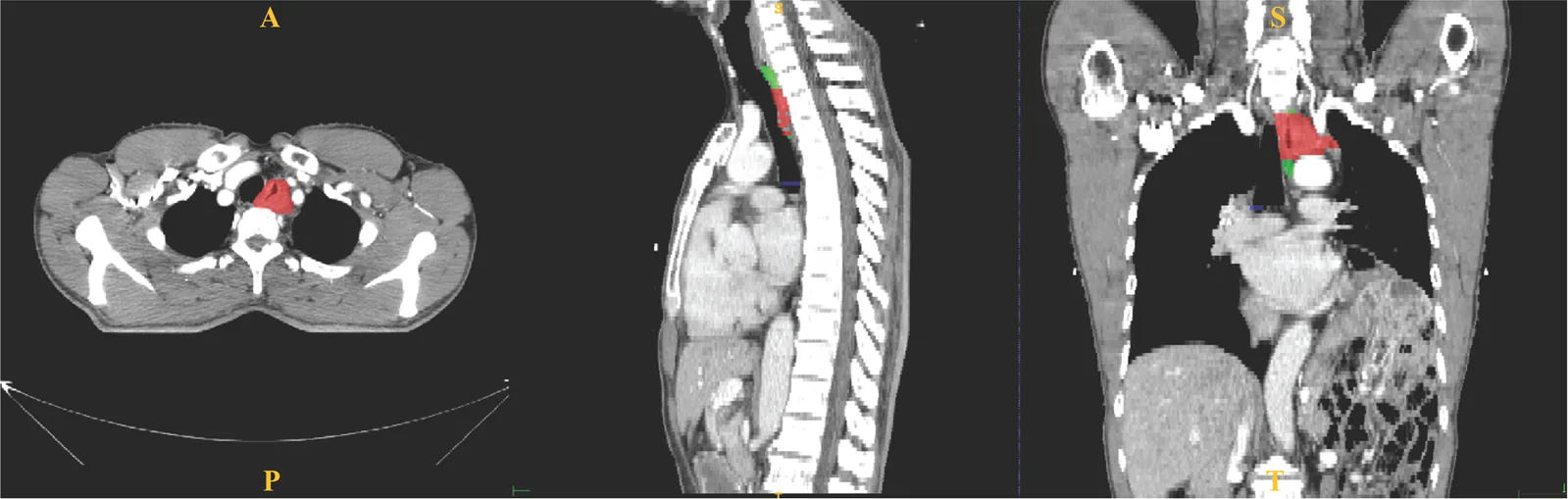
Examples of CT images and tumor lesion contours. Axial, coronal, and sagittal CT images of a patient with esophageal cancer. The red mark represents the lesion of esophageal cancer and the green mark represents the normal esophagus above and below the lesion of esophageal cancer.

**Fig. (2):**
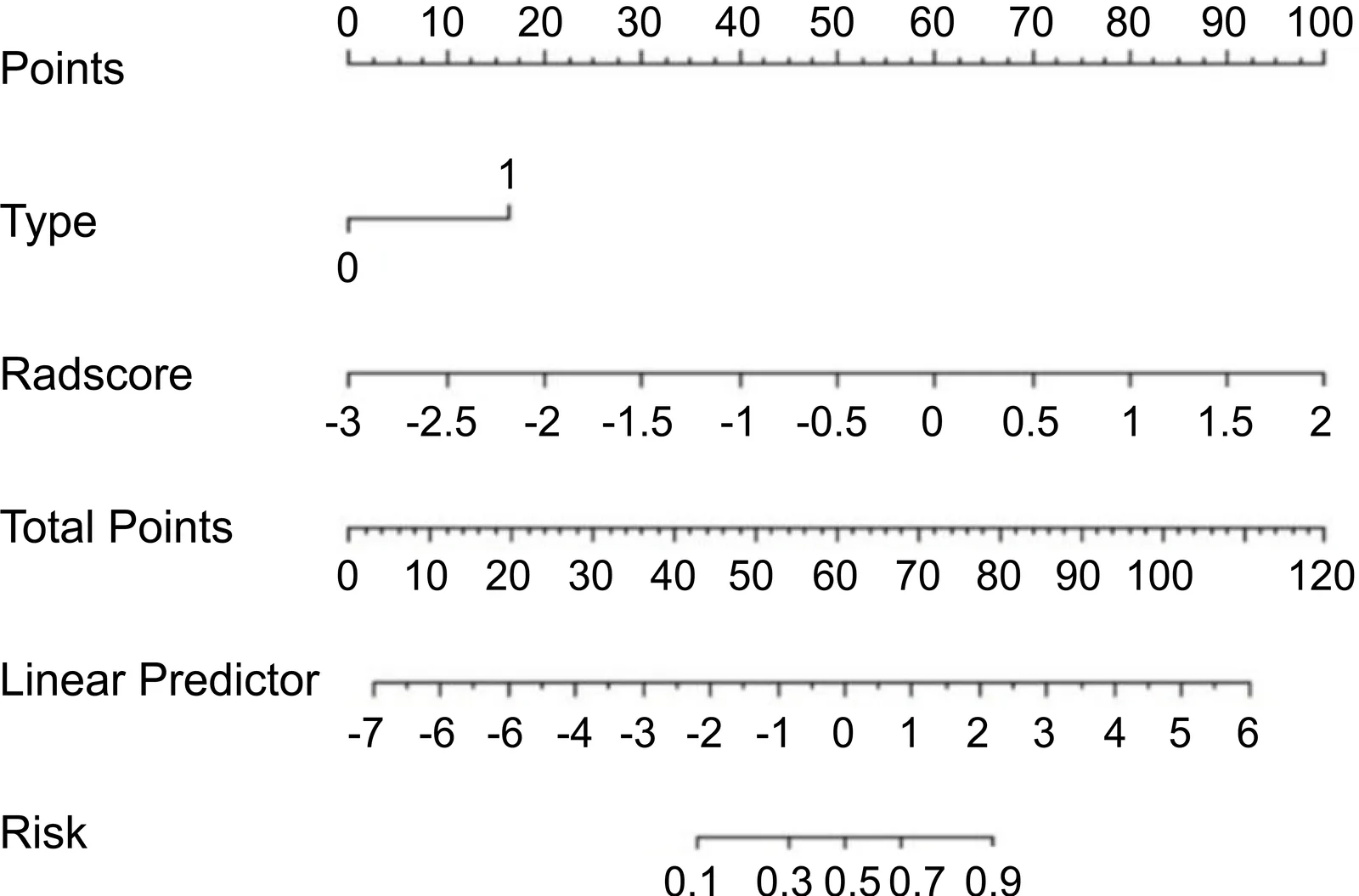
Nomogram. By integrating clinical factors and radiomics characteristics, a nomogram is constructed to predict the likelihood of esophagotracheal fistula occurring.

**Fig. (3):**
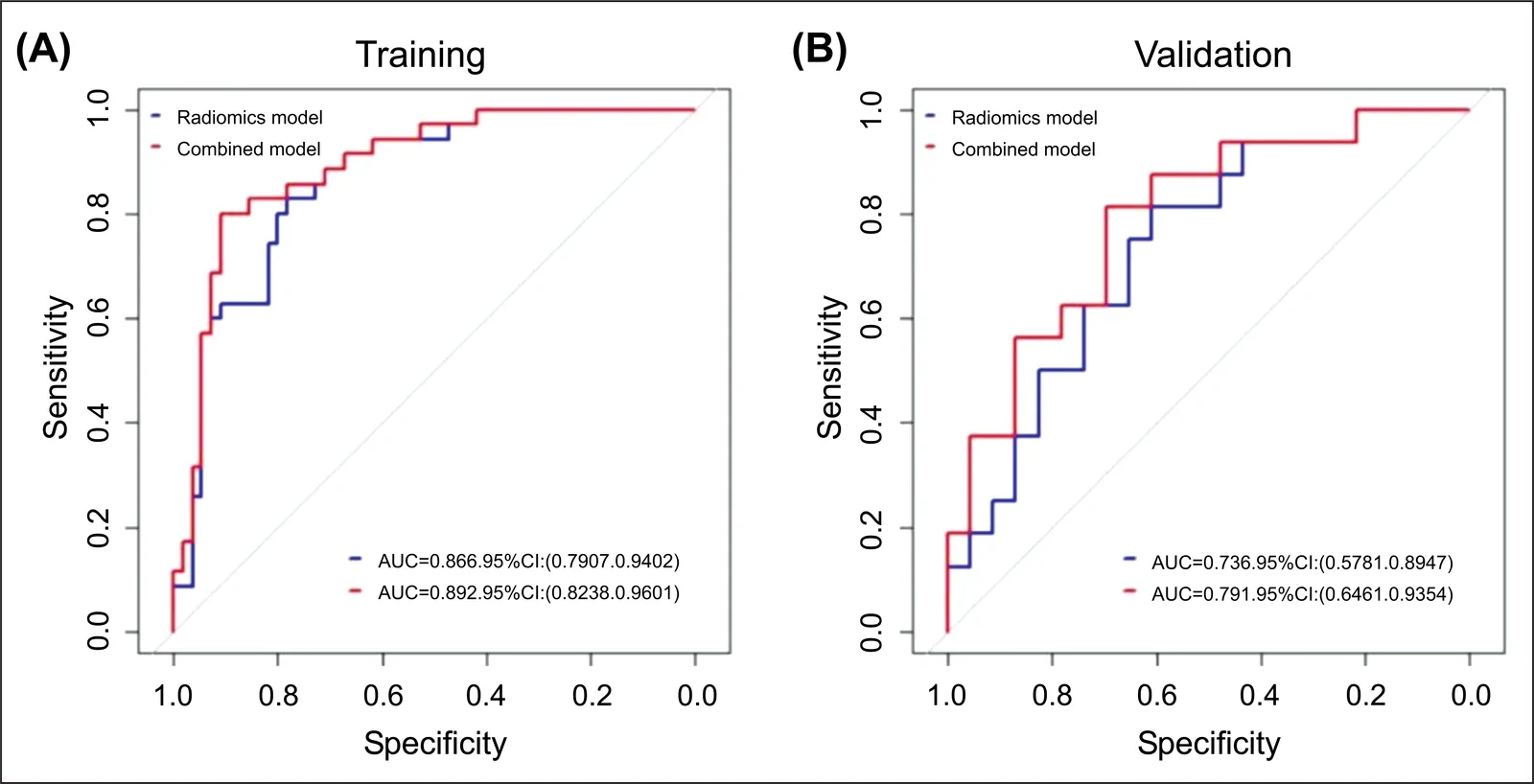
ROC curves of radiomics models and combination models. (**A**): Training cohort; (**B**): Validation cohort.

**Fig. (4):**
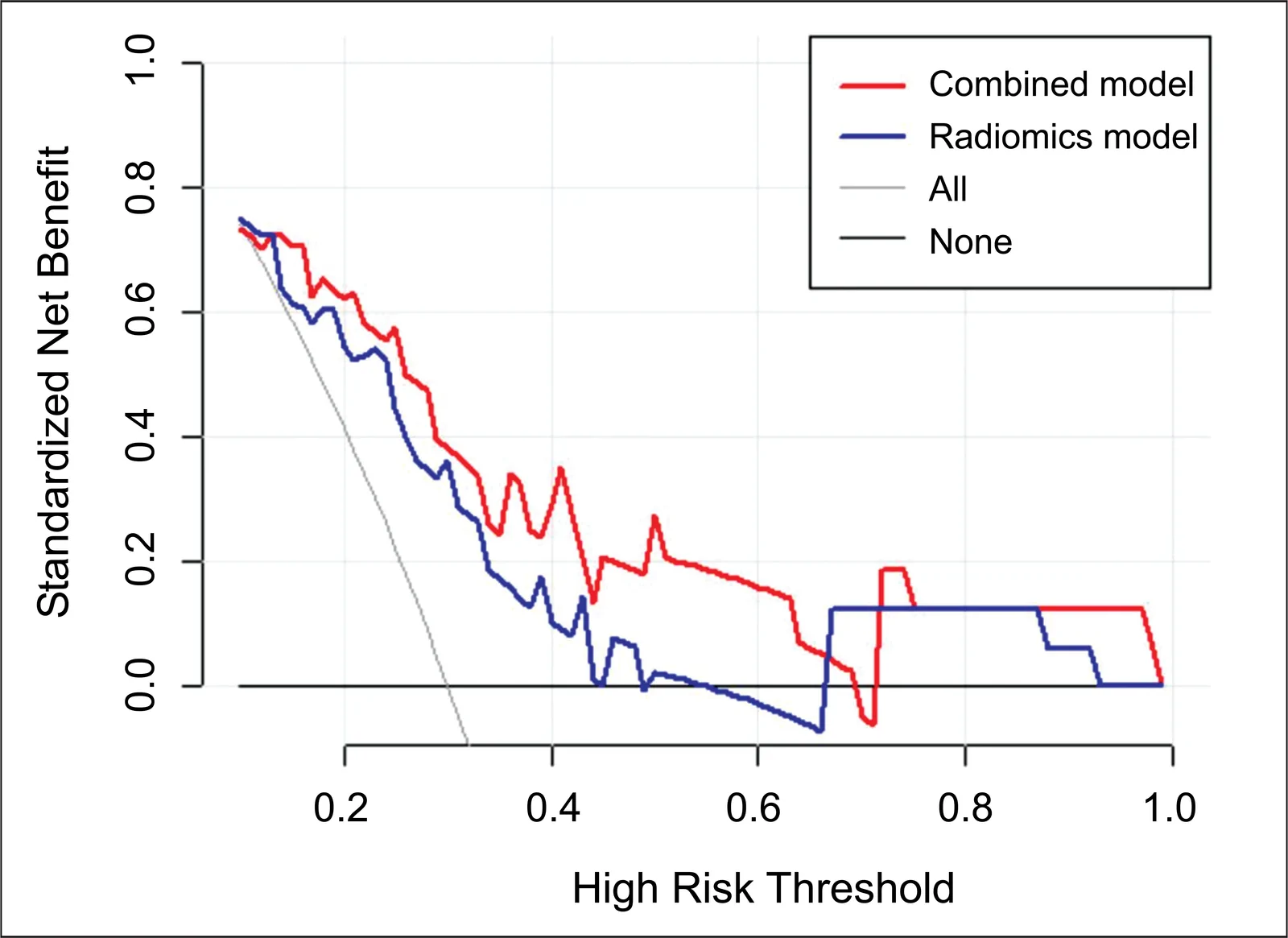
Decision Curve Analysis (DCA). Decision curve analysis demonstrated favorable clinical utility of the combined model.

**Table 1:** Clinical feature.

Clinical feature	
Age/year	65.7(31-85)
Radiation dose/Gy	51.8(40-70)
Gender	
Male	71(92.2)
Female	6(7.8)
Tumor Location	
Upper	28(36.7)
Middle	25(32.2)
Lower	24(31.1)
Chemotherapy	
Yes	52(67.8)
No	25(32.2)
Hypertension	
Yes	21(26.7)
No	56(73.3)
Diabetes	
Yes	10(13.3)
No	67(86.7)
Smoking	
Yes	26(33.3)
No	51(66.7)
Radiotherapy type	
IMRT	57(74.4)
2DRT	20(25.6)

**Abbreviation:** Two-dimensional radiotherapy (2DRT), Intensity modulated radiotherapy (IMRT).

**Table 2:** Univariate and multivariate analyses.

**Variable**	**Univariate analyses**			**Multivariate analyses**		
	**OR**	**95% CI**	**p**	**OR**	**95% CI**	***p***
Age	0.965	(0.926,1.041)	0.301			
Radiation dose	1	(0.999,1.000)	0.044*	1	(0.999,1.000)	0.402
Gender	0.314	(0.031,2.530)	0.374			
Tumor location	0.872	(0.314,2.547)	0.44			
Chemotherapy	0.783	(0.311,1.995)	0.672			
Hypertension	0.844	(0.336,2.241)	0.767			
Diabetes	0.897	(0.285,3.128)	0.845			
Smoking	0.521	(0.234,1.233)	0.138			
Radiotherapy type	0.176	(0.045,0.562)	<0.001*	0.164	(0.053,0.671)	0.004*
Radiomics score	9.665	(3.751, 26.513)	<0.001*	11.532	(3.612, 32.542)	<0.001*

**Abbreviations:** odds ratio (OR),confidence interval (CI).

## Data Availability

The data and supporting information are available in the article.

## LIST OF ABBREVIATIONS

ETF: Esophagotracheal Fistula
RT: Radiation Therapy

## STANDARDS OF REPORTING

STROBE guidelines were followed.
